# Post-traumatic stress disorder following COVID-19 pandemic among medical students in Riyadh: a cross-sectional study

**DOI:** 10.1186/s43045-021-00127-3

**Published:** 2021-07-23

**Authors:** Zainab Ifthikar, Saima Sajjad Fakih, Saumy Johnson, Johnson Alex

**Affiliations:** 1 College of Medicine, Alfaisal University, Riyadh, Kingdom of Saudi Arabia; 2 Department of Respiratory Therapy, Inaya Medical Colleges, Riyadh, Saudi Arabia; 3 Clinical Psychology, College of Medicine, Dar Al Uloom University, Riyadh, Saudi Arabia

**Keywords:** COVID-19, IES-R, Pandemic, Medical students, Post-traumatic stress disorder

## Abstract

**Background:**

In recent times, COVID-19 has been recognized as a public health emergency and thus far, most papers published on it are focused only on the clinical characteristics of infected patients. This pandemic has also made phenomenal emotional impact among the young and the old. We aimed to find out the impact of the COVID-19 pandemic on the psychological well-being of medical students in a University at Riyadh.

**Results:**

There were 309 participants in the study. Out of them 44% did not have PTSD, 29% had score more than 37 which might contribute to immune suppression, in 18.4% PTSD was a clinical concern and 8.6% had probable PTSD. Female participants were the majority in the group and they also had higher chance of having consequences than the male counterparts (*P* < 0.001). Avoidance score between male and female gender was significantly different.

**Conclusion:**

COVID-19 pandemic has not just affected the physiological functioning of the affected individuals but also has had a probable post-traumatic stress disorder among young college students. Screening for psychological well-being and the treatment for PTSD is imperative in college, school and general population.

## Background

The novel corona virus (SARS-CoV-2) causing COVID was first identified in Wuhan, China, and then spread across many countries through droplet and contact transmission. It has changed the social life of people across the world and has added new isolation measures and quarantine to their life [1]. This isolation is known to affect their mental health due to the loss of a true human connection and surging feelings of stress and anxiety during times of uncertainty.

The public health event it caused has led to delays in starting schools and universities and a switch to online modes of teaching [1]. SARS-CoV-2 virus reached Saudi Arabia in March 2020; a state of alert was declared, slowly leading to nationwide curfew. The Alfaisal University campus was closed in March, online classes resumed shortly, and the closure continued till the end of the spring semester in May 2020.

The impact of COVID-19 worldwide includes not only increased fatality rates but also a wide plethora of both psychological and economic impacts that are now coming to light. The mass fear of COVID-19, termed “Coronaphobia,” along with the forced quarantine, has increased levels of anxiety, hoarding behaviors, and PTSD among the general population [2].

The first association between viral epidemics and psychological distress was made over a 100 years ago during the 1918 Spanish flu pandemic that yielded psychiatric complications [3].

In recent times, COVID-19 has been recognized as a public health emergency and thus far, most papers published on it are focused only on the clinical characteristics of infected patients. However, this pandemic has also demanded people to show phenomenal emotional resilience in the solitude of their homes which can take a toll on their mental health [4].

Negative mental health consequences were observed in medical students as well and can be attributed to isolation from friends and family, experiencing doubts about the disease and having to manage medical school learning during the lockdown [5].

Mental health is affected by a multitude of factors including, but not limited, to our physical health, self-perception, relationships, and social connections. It is therefore inevitable to overlook the impact of a closure of innumerable industries, such as education, finance, entertainment, and service sectors, on the fragile mental health of people belonging to vulnerable subsets of the population.

Furthermore, corona positive patients are now considered a stigma [2]. Internet and social media consumption are fueling safety seeking and hoarding behaviors [6] and affective temperament traits are being recognized in response to the outbreaks. In Italy, anxious, cyclothymic, and depressive temperaments were adopted as modalities of behavior. While anxious behaviors suggested a protective nature, cyclothymic and depressive temperaments indicated worry, negativity, and self-doubt with an enhanced desire for social connection [3].

Other stressors include inadequate supplies, fears of catching the infection, and inadequate information. The most at risk were quarantined medical staff with complaints of increased exhaustion, anxiety, poor concentration, and indecisiveness. In addition, coping mechanisms such as rumination, self-blame, and denying/disengagement were associated with increased psychological complaints [7].

Investigations of the symptoms of psychological determent were described as being very few in the beginning of global alert; people with chronic diseases who were young accounted for more symptoms, and later on there were increased symptoms being detected preceding stay home announcement was given in some countries [8, 9].

On the other hand, a study comparing undergraduate students who had been quarantined found no significant difference in mental health problems when compared to those who had not. Possible reasons include fewer responsibilities, and younger and lesser susceptibility to the infection [7].

Another study demonstrated that medical students believed quarantine did not affect their learning and psychological well-being. Though, some students showed a sense of emotional detachment from friends and family and claimed that this decreased their overall study performance. The main causes of deterioration of mental health were financial losses, fear, frustration, and inadequate information [5].

Overall, stress experienced by young students can be due to the uncertainties of a new learning environment, and the need to adapt to the absence of in-person lectures [8]. Out of town students expressed worry for not only their educational gains but also the safety of their families once they return home [10].

The ongoing COVID-19 pandemic may perhaps leave us all in turmoil to deal with even long after the actual pandemic has culminated. Addressing the issues, it is creating along the way can help mitigate risks for our communities one by one. Coping with stress in a healthy way will make community stronger, it will allow us to combat similar pandemics in the years to come and also allow us right now to provide coping mechanisms for people in social isolation, help address overwhelming media consumption, and improve adherence to guidelines without the associated stress.

All things considered, after the end of quarantine, behavioral changes such as avoidance behaviors and vigilant handwashing were recorded for several months in certain places. Financial loss and increased stigma contributed to the long-term effects of the quarantine on mental health. General education and proper explanations on the rationale of quarantine is thought to reduce this stigma [7].

In contrast to the effect brought about by the social scene, SARS-CoV-2 is presumed to have direct pathological effects too due to it being a multiorgan system damaging virus. It may also infect the brain and trigger the immune system affecting brain function and consequently individual’s psychological health [11].

Through this study, we will be able to determine the impact of the COVID-19 pandemic on the psychological well-being of medical students in Alfaisal University, Riyadh, Saudi Arabia. Formulating this association will better equip us to combat similar consequences in the future if the social isolation continues into the upcoming semester. Furthermore, we will be able to assess the symptoms of post-traumatic stress disorder (PTSD) in the context of COVID-19 among medical students. The IES-R scale [12, 13] used in this study is specifically designed to measure the symptoms of PTSD using three parameters: intrusion, avoidance, and hyperarousal. The presence of these symptoms will help further investigate the specific consequences that were profoundly brought about by the pandemic and guide us toward formulating appropriate timely interventions.

## Methods

This research was a cross-sectional study which was conducted at Alfaisal University from June 16, 2020, to August 18, 2020. Subjects were students enrolled in Alfaisal College of Medicine during the 2019-2020 academic year from year 1 to year 5.

The target population was the undergraduate students from year 1 to year 5 from the college of medicine at Alfaisal University, which is approximately 1094 students, for the academic year 2019-2020.

Based on the previous study conducted among medical students in Riyadh, a sample size of 384 was calculated at 95% level of significance and 5% allowable error. According to a study conducted previously at Alfaisal University, the prevalence of anxiety was high and reported as being 63% [14]. Another study conducted elsewhere claimed that the prevalence of severe stress among medical students constituted 33.8% and concluded that the mean stress scores were 26.03 ± 9.7 [15].

This study was aimed at investigating the impact of the COVID-19 pandemic on the mental health of medical students which is also reflected by the level of anxiety. Anxiety, as backed up by our literature review, is defined as a key parameter in influencing mental health. Hence, the proportion of the population estimated to have anxiety was derived as 63% and sample size was planned as 358.

The questionnaire used was the Impact of Event Scale-Revised (IES-R) questionnaire which is a self-report questionnaire. This tool is being used in the diagnosis of the psychological impact of a traumatic event. It consists of 22 questions for which the responses will be recorded on a five-point Likert scale.

The questionnaire had a section of general sociodemographic questions such as age, gender, and academic year without any information that might disclose the identity of the participant. The main IES-R questionnaire consists of three subscales [12, 13]. (1) Intrusion: assessing intrusive thoughts, nightmares, and dissociative feelings. (2) Avoidance: lack of responsiveness to situations and ideas. (3) Hyperarousal: anger, lack of concentration, and increased startle. IES-R is a 22-itemscale that comprises of three subscales that quantify the mean avoidance, intrusion, and hyperarousal [14]. Responses to each item are evaluated from 0 to 4, where 0 indicates Not at all and 4 Extremely. IES-R total score is divided into Normal which is from 0 to 23, Mild from 24-32, Moderate from 33–36, and Severe psychological impact more than 37. IES-R English version was used in this study and it was previously used conducting researches among Saudi student population.

Study was conducted after the ethical and IRB approval from the University and an informed consent was obtained from all participants prior to filling out the survey.

### Statistical analysis

Descriptive statistics is given as mean ± SD and frequency or %. Calculation of IES-R score was done in Microsoft Excel after the data was transferred from Google sheet. Scores for the avoidance, intrusion, and hyperarousal subscales were calculated [12, 13]. Mean of question item numbers 5, 7, 8, 11, 12, 13, 17, and 22 were taken to provide the avoidance subscale score. Mean of question item numbers 1, 2, 3, 6, 9, 14, 16, and 20 were taken to provide the intrusion subscale score. Mean of question item numbers 4, 10, 15, 18, 19, and 21 were taken to provide the hyperarousal subscale score. The sum of the mean values from these three subscales was then used to calculate the total mean IES-R score. The IES-R scoring ranges from 0 to 88. Any score above 24 is associated with a meaningful result. The consequence of each score was then recorded as a comment as per the following table:

**Table:** 

Score	Consequence
24 or more	**PTSD is a clinical concern** [16]. Individuals who have such high score who does not qualify full PTSD might be having a partial PTSD or few of the symptoms.
33 and above	This value is the recommended cutoff for **probable diagnosis of PTSD** [17].
37 or more	This increased level can suppress the immunity (even 10 years after an impact event) [18].

Chi square test was done to see the association. Data was analyzed in SPSS 23.1 version (IBM SPSS Statistics). *P* value of < 0.05 was considered significant.

## Results

This was a cross sectional study conducted in 309 participants. An online questionnaire was sent to the participants in the Google form. Response rate was around 65%. Impact of Event Scale-Revised questionnaire which is a standard questionnaire was used for collecting the information for this research. There were 225 female and 84 male participants in this study. Academic year distribution and gender distribution is given in Table 1. Academic year was noted and 83 (26.8 %) were from year 1, 53 (17.1%) from year 2, 66 (21.3%) from year 3, 48 (15.5%) from year 4, and 59 (19.0%) from year 5.

**Table 1:** Demographic details

Variables	Frequency (%)
**Gender**	
Female	225 (72.6%)
Male	84 (27.1%)
**Academic year**	
1	83 (26.8 %)
2	53 (17.1%)
3	66 (21.3%)
4	48 (15.5%)
5	59 (19.0%)

**Fig. 1:**
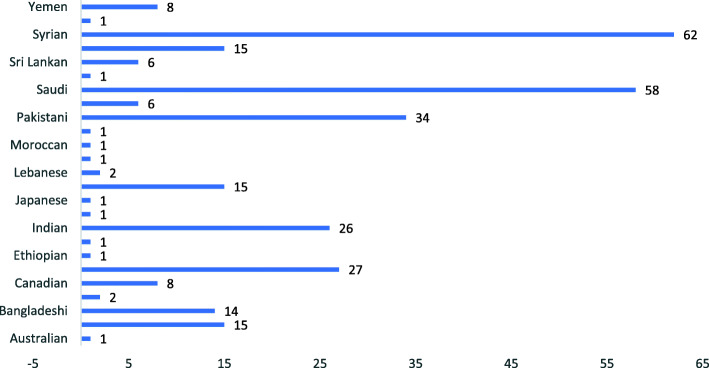
Distribution of the nationalities among the participants

Figure 1 shows the distribution of the nationality among the participants. Syrian nationalities were the majority followed by Saudi nationals and then Pakistan nationals. Other nationalities made small percentage of the participants.

**Fig. 2:**
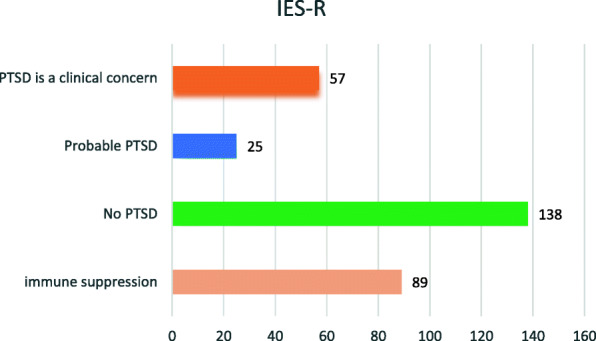
Impact of Event Scale-Revised in the participants

Impact of Event Scale-Revised (IES-R) score was evaluated and categorized in to four categories according to the consequence; post-traumatic stress disorder (PTSD) is a clinical concern, probable PTSD, no PTSD, and immune suppression. As shown in Fig. 2, 44% did not have PTSD, 29% had score more than 37 which might contribute to suppression of immune system functioning. In 18.4%, PTSD was a clinical concern and 8% had probable PTSD. IES-R consequence score was assessed and median score was 26 with IQR of 15, 39 respectively.

**Fig. 3:**
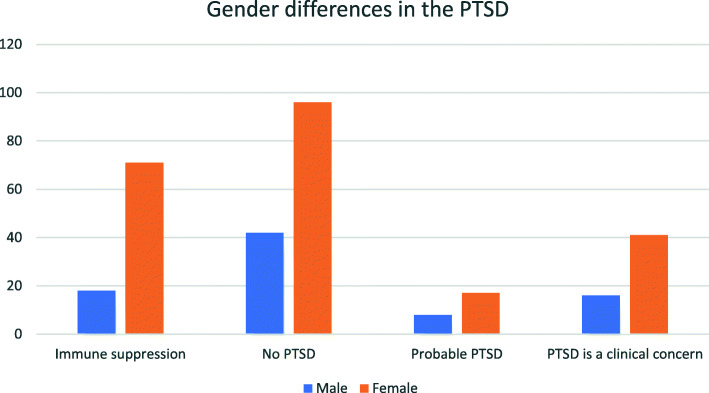
Gender differences in the IES-R consequences

Chi square test was done to see the association between gender and the IES-R consequences, which showed significant differences between the gender and the category of PTSD. Gender differences in the IES-R consequences are given in Fig. 3. Female participants were the majority in the group and they also had higher chance of having consequences than the male counterparts (*P* < 0.001).

Analysis of IES-R score revealed that there was difference only in the avoidance score between male and female gender in terms of their difference in IES-R score. Table 2 shows participant’s IES-R score according to the gender difference.

**Table 2:** Participant’s IES-R score according to the gender difference

IES-R sub scales	***N***	Mean rank	Sum of ranks	***P*** value
Intrusion				
Male	84	155.24	34,928.50	> 0.05
Female	225	154.36	12,966.50	
Avoidance				
Male	84	139.90	11,752.00	0.03
Female	225	160.20	36,046.00	
Hyperarousal				
Male	84	141.06	11,849.00	> 0.05
Female	225	160.20	36,046.00	

## Discussion

This prospective cross-sectional study among medical students in Saudi Arabia had 72% female participants and 28% male participants. Participants were from different countries who came to Saudi Arabia to pursue their education. Highest number of participants was from Syria, second highest from Saudi Arabia, and third highest from Pakistan.

This study used IES-R to evaluate PTSD and its test-retest reliability (*r* = 0.79 to 0.89) and internal consistency (Cronbach’s *α* = 0.78 to 0.82) has been proved to be acceptable.

This study was designed based on previous studies which found increased risk of psychopathologies and stress-related disorders, as well as a high rate of PTSD symptoms [19–24]. A research conducted in the Liaoning Province in China highlighted that 52.1% of their participants had feelings of apprehension due to the pandemic [9]. In general, females were identified as high-risk groups for mental health [9] and males were deemed less likely to develop mental health problems [3]. Children who were quarantined showed 4 times higher post-traumatic stress scores when compared to children who were not quarantined [7]. In our study, 29% had score more than 37 which might contribute to suppression of immune system functioning, in 18.4% PTSD was a clinical concern and 8% had probable PTSD. A study done by A.H. Khan et al. showed that a considerable percentage of population are at high risk of psychological consequences during COVID-19 outbreak [25]. In a study done in Bangladesh, a large number of participants had depression, anxiety, and stress 4 months after the COVID-19 outbreak [26]. A study done among medical students in Pakistan showed that they feel detached emotionally from the family, fellows, and friends and they had decreased overall work performance and study period. A study done in Saudi Arabia showed that during the initial days of COVID-19 pandemic in Saudi Arabia, one-fourth of the individuals had moderate-severe impact on their psychological well-being [27, 28].

A cutoff value of 33 is used to identify the presence of probable PTSD [29]. In this study, there were 146 participants out of 309 (47%) who were having a PTSD event. COVID-19 pandemic has affected the young college student population in a great extent that is shown as 47% of the PTSD events among the study population.

Gender differences in the PTSD event were evaluated. It was seen that female participants had probable PTSD more than males in this study and it was statistically significant. Breslau N et al. found that PTSD develops more in female than male after the individual is exposed to traumatic event [30]. This shows the impact of this pandemic on female college students than male and the support system should be provided to take care of this situation during these unexpected and stressful situations.

IES-R scale looks at the different aspects of intrusion, avoidance, and hyperarousal. In this study, the sub scale intrusion and hyperarousal was not statistically different between male and female but avoidance (numbing of responsiveness, avoidance of feelings, situations, and ideas) was significantly different between male and female participants. There was no correlation between nationality and the IES score. A study that was conducted all around the world through an online survey showed that depression and stress scoring of the participants were very high especially among Indian and Pakistani nationals [31].

### Limitation of the study

There are few limitations of the study; firstly, the survey was done online which may be less effective than face to face. Secondly, other psychological screening tools were not used.

Further research is warranted in this topic with bigger sample size.

## Conclusion

COVID-19 pandemic has affected medical students to a greater extend. The post-traumatic stress disorder (PTSD) symptoms that the participants showed are evidences that this pandemic has done serious impact on the psychological well-being of young people especially medical students. Continued psychological support and timely interventions are imperative to tackle this disastrous situation.

### Recommendations

This cross-sectional study throws light in to the need for the availability and access of a professional psychological help at medical colleges especially in this period of pandemic where students are at greater amount of stress. Further research is warranted in this topic with bigger sample size.

## Data Availability

The datasets used and/or analyzed during the current study are available from the corresponding author on reasonable request.

## Abbreviations

PTSD: Post-traumatic stress disorder
IES-R: Impact of Event Scale-Revised
